# A machine learning-based quantitative model (LogBB_Pred) to predict the blood–brain barrier permeability (logBB value) of drug compounds

**DOI:** 10.1093/bioinformatics/btad577

**Published:** 2023-09-15

**Authors:** Bilal Shaker, Jingyu Lee, Yunhyeok Lee, Myeong-Sang Yu, Hyang-Mi Lee, Eunee Lee, Hoon-Chul Kang, Kwang-Seok Oh, Hyung Wook Kim, Dokyun Na

**Affiliations:** Department of Biomedical Engineering, Chung-Ang University, Seoul 06974, Republic of Korea; Department of Biomedical Engineering, Chung-Ang University, Seoul 06974, Republic of Korea; Department of Biomedical Engineering, Chung-Ang University, Seoul 06974, Republic of Korea; Department of Biomedical Engineering, Chung-Ang University, Seoul 06974, Republic of Korea; Department of Biomedical Engineering, Chung-Ang University, Seoul 06974, Republic of Korea; Division of Pediatric Neurology, Department of Pediatrics, Severance Children’s Hospital, Yonsei University College of Medicine, Epilepsy Research Institute, Seoul 03722, Republic of Korea; Department of Anatomy College of Medicine, Yonsei University, Seoul 03722, Republic of Korea; Convergence Drug Research Center, Korea Research Institute of Chemical Technology, Daejeon 34114, Republic of Korea; Department of Bio-integrated Science and Technology, College of Life Sciences, Sejong University, Seoul 05006, Republic of Korea; Department of Biomedical Engineering, Chung-Ang University, Seoul 06974, Republic of Korea

## Abstract

**Motivation:**

Efficient assessment of the blood–brain barrier (BBB) penetration ability of a drug compound is one of the major hurdles in central nervous system drug discovery since experimental methods are costly and time-consuming. To advance and elevate the success rate of neurotherapeutic drug discovery, it is essential to develop an accurate computational quantitative model to determine the absolute logBB value (a logarithmic ratio of the concentration of a drug in the brain to its concentration in the blood) of a drug candidate.

**Results:**

Here, we developed a quantitative model (LogBB_Pred) capable of predicting a logBB value of a query compound. The model achieved an *R*^2^ of 0.61 on an independent test dataset and outperformed other publicly available quantitative models. When compared with the available qualitative (classification) models that only classified whether a compound is BBB-permeable or not, our model achieved the same accuracy (0.85) with the best qualitative model and far-outperformed other qualitative models (accuracies between 0.64 and 0.70). For further evaluation, our model, quantitative models, and the qualitative models were evaluated on a real-world central nervous system drug screening library. Our model showed an accuracy of 0.97 while the other models showed an accuracy in the range of 0.29–0.83. Consequently, our model can accurately classify BBB-permeable compounds as well as predict the absolute logBB values of drug candidates.

**Availability and implementation:**

Web server is freely available on the web at http://ssbio.cau.ac.kr/software/logbb_pred/. The data used in this study are available to download at http://ssbio.cau.ac.kr/software/logbb_pred/dataset.zip.

## Graphical Abstract

**Graphical Abstract:**
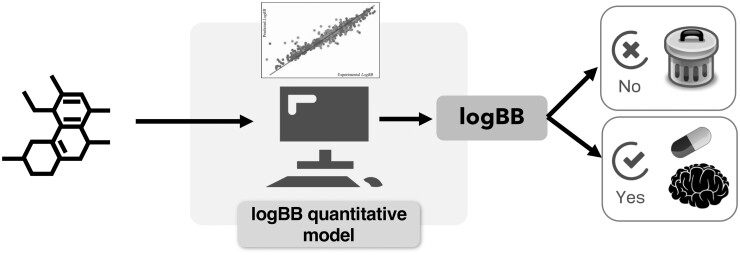

## 1 Introduction

The blood–brain barrier (BBB) is a highly selective semi-permeable membrane composed of endothelial cells. The BBB regulates the transport of molecules from blood vessels to the central nervous system (CNS) (Bradbury 1993), and the tightly selective permeability enables to maintain homeostasis of the brain microenvironment, and protects the CNS from damage by harmful substances (Abbott *et al.* 2010).

CNS diseases are the second most common following cardiovascular diseases (Vilella *et al.* 2015). The lower success rate of the CNS drugs (8%) than that of the cardiovascular drugs (20%) is mainly due to the BBB since most small-molecule and macromolecule drugs are not able to cross through the BBB into the brain (Chen and Liu 2012, Gao *et al.* 2013, Valentini *et al.* 2019). Therefore, BBB permeability of CNS drugs should be improved to elevate the success rate in CNS drug discovery (Di *et al.* 2013).

Various *in vivo* and *in vitro* experimental assays have been developed to measure the BBB permeability of molecules: a logarithmic ratio of the concentration of a drug in the brain to its concentration in the blood (logBB) (Abbott 2004, Carpenter *et al.* 2014, Ciura and Dziomba 2020). *In vitro* methods such as parallel artificial membrane permeability assay (PAMPA) and immobilized artificial membrane (IAM) typically use cultured brain tissue cells or artificial membranes to measure a drug concentration on each side (Reichel *et al.* 2003, Carrara *et al.* 2007, Mensch *et al.* 2009). Though *in vitro* methods have advantages in performing experiments in parallel and are suitable for drug screening, none of the methods can reproduce *in vivo* environments and, thus, *in vitro* logBB values are often not consistent with *in vivo* logBB values (Colquitt *et al.* 2011). On the contrary, *in vivo* methods using living animals are appropriate to obtain real logBB values, but they are more difficult to conduct as well as time-consuming and laborious, and thus are not suitable for large-scale experiments (Srinivasan *et al.* 2015, Valentini *et al.* 2019).

Due to the experimental difficulties, computational methods have been introduced to estimate the BBB permeability of drug candidates (Kumar *et al.* 2013; Radan *et al.* 2022). Early prediction models were mainly qualitative and predicted whether a query compound was BBB-permeable or nonpermeable (Muehlbacher *et al.* 2011). Several machine learning algorithms including random forest (RF) (Svetnik *et al.* 2003), support vector machine (SVM) (Ghorbanzad’e and Fatemi 2012), genetic algorithm (Shen *et al.* 2008), and artificial neural network (ANN) (Jung *et al.* 2007) have been used to develop BBB permeability classification models (Gerebtzoff and Seelig 2006, Guerra *et al.* 2008, Mehdipour and Hamidi 2009, Martins *et al.* 2012, Suenderhauf *et al.* 2012, Wang *et al.* 2018, Singh *et al.* 2020, Tang *et al.* 2022). In a recent study, a BBB classification model was developed based on Light Gradient Boosting Machine (LightGBM), a gradient-boosting framework based on decision tree algorithms, with 7162 compounds and achieved a high area under the curve (AUC) value of 0.94 (Shaker *et al.* 2021).

Recently, the demand for quantitative BBB permeability models has been increasing to predict the permeability of drug candidates to cross BBB (Muehlbacher *et al.* 2011). Several quantitative models have been developed with the logBB values of compounds. However, since the publicly available logBB dataset is very limited, it is difficult to develop a high-performance quantitative model using a small dataset. Therefore, out of several published quantitative models, only few are publicly accessible (Platts *et al.* 2001, Bayat *et al.* 2011, Muehlbacher *et al.* 2011, Shin *et al.* 2021). One of the publicly accessible models for quantitative prediction is PreADMET (Lee *et al.* 2004). It is an online web server for the quantitative prediction of drug properties, developed by Lee *et al.*, in 2004 based on ANN trained with the 2D descriptors calculated by TOPOMOL (Lee *et al.* 2004, Polyakova *et al.* 2006). Another available model is ADMET Prediction Service developed by Dyabina *et al.* (2016). It was trained based on ANN with the logBB values of 529 compounds. In a recent study by Ciura *et al.* (2020), multi-linear regression (MLR) and SVM were developed with known logBB values of only 45 marketed drugs. They used 30 of the drugs for model training and 11 for testing. The models achieved an *R*^2^ score of 0.69 by SVM model and 0.76 by MLR model on the training dataset. When applied to the test dataset, *R*^2^ was over > 0.9. The abnormally high *R*^2^ score on the test dataset might be due to the extremely small amount of data. Wang *et al.*, assembled a dataset of 439 logBB values (341 for training and 98 for validation) and developed three machine learning models based on RF, SVM, and k-nearest neighbor (*k*NN) using 192 2D descriptors calculated by Molecular Operating Environment (MOE) (Wang *et al.* 2015). Then, they developed a consensus model that averages the predicted scores generated from the three machine learning models. The consensus model attained an *R*^2^ of 0.52 on validation dataset. Liu *et al.* (2001) developed a quantitative structure activity relationship (QSPR) models to evaluate the BBB penetration. The authors used a dataset of 112 compounds with experimentally determined BBB penetration and calculated various molecular descriptors using Dragon software. They used MLR and partial least squares regression to develop QSPR models. The best model achieved an *R*^2^ of 0.70 on validation dataset. Wu *et al.*, proposed an ANN model to predict the BBB permeability of drug-like compounds (Wu *et al.* 2021). The model used a group contribution method to estimate the molecular descriptors and was trained on a dataset of experimentally measured logBB values of 255 compounds. The model achieved a prediction accuracy with a relative error of 0.810 and root mean square error (RMSE) of 0.236 on an external validation dataset (40 compounds).

Here, we aimed at developing a quantitative BBB permeability prediction model with a larger dataset and thereby having a higher accuracy. To the best of our knowledge, we compiled the largest logBB dataset from various literature (Platts *et al.* 2001, Fu *et al.* 2005, Bayat *et al.* 2011, Muehlbacher *et al.* 2011, Carpenter *et al.* 2014, Shin *et al.* 2021, Tang *et al.* 2022) and used a gradient boosting machine learning algorithm (LightGBM) for model construction (Zhang *et al.* 2019). Our constructed model (LogBB_Pred) showed an *R*^2^ of 0.61 and mean square error (MSE) of 0.36, which were better than publicly available quantitative BBB models when evaluated on a test dataset. Our model is freely accessible *via*  http://ssbio.cau.ac.kr/software/logbb_pred/ for practical use and we believe that our model would be useful in early high-throughput screening of CNS drugs and would increase the success rate in CNS drug development.

## 2 Materials and methods

### 2.1 Dataset collection and preprocessing

The size and quality of datasets greatly impact the performance of the prediction models trained by machine learning algorithms (Chen *et al.* 2021). For a better performance, we compiled the largest dataset, to the best of our knowledge, of the experimentally measured 1276 logBB values from the literature (Platts *et al.* 2001, Fu *et al.* 2005, Bayat *et al.* 2011, Muehlbacher *et al.* 2011, Carpenter *et al.* 2014, Shin *et al.* 2021, Tang *et al.* 2022). To avoid bias in the dataset leading to a biased or overfitted prediction model, similar chemical compounds were discarded based on Tanimoto similarity with a cutoff of 0.85 (Bajusz *et al.* 2015, Macomber *et al.* 2015). For the similarity calculation, chemical compounds were represented in the format of simplified molecular-input line-entry system (SMILES) (Weininger 1988) and their fingerprints were calculated by Dragon software (Mauri *et al.* 2006). Tanimoto similarity was calculated based on the fingerprints of compounds. Consequently, the final dataset contained 913 logBB values ranging from −2.69 to 1.7. The equation for Tanimoto similarity is:


(1) $$T_{\left(a,\;b\right)}=\;\frac{N_{c}}{N_{a}+N_{b}-N_{c}}.$$

*T* denotes Tanimoto similarity between molecules *a* and *b*, where *N_a_* and *N_b_* represent the numbers of *on* bits in the molecules *a* and *b*, and *N_c_* denotes the number of bits that are *on* in both molecules.

For feature preparation, the physical and chemical properties of chemical compounds were calculated from the chemical structures represented in the SMILES format. Specifically, the properties (1650 2D/3D molecular descriptors) including eccentric connectivity index (Sharma *et al.* 1997) and charged partial surface area (Stanton and Jurs 1990) were calculated using a publicly available tool, Mordred, which is a recently published molecular descriptor calculator (Grisoni *et al.* 2018, Moriwaki *et al.* 2018).

After removing descriptors with missing values, the resulting dataset contained 1164 informative molecular descriptors for each compound. Since there might be redundant features, we filtered out such features by Pearson’s correlation coefficient (PCC) between features (Thakkar *et al.* 2021). If two features are redundant, only one with lower PCC with logBB was discarded. To find an optimal feature set, different training datasets were constructed with different coefficient thresholds from 0.1 to 0.9. After filtering, features were normalized using standard scaling technique (Raju *et al.* 2020). The datasets were used for cross-validation and an optimal threshold was determined.

In order to validate model performance, we collected additional 109 compounds (Hou and Xu 2003). Compounds that showed a Tanimoto similarity >0.85 with those in the training dataset were discarded, which resulted in a total of 27 unique compounds. These compounds were used as an independent test dataset for external validation. For the evaluation of our model as a classification model, we also collected binary data of compounds (BBB-permeable and BBB-nonpermeable) from MedChemExpress (https://www.medchemexpress.com/).

### 2.2 Model construction and evaluation

In this study, we used LightGBM algorithm to develop a regression model to predict BBB permeability (logBB value) (Zhang *et al.* 2019). LightGBM is an advanced method of gradient boosting decision tree and is known to perform better than other decision tree learning algorithms (Friedman 2001, Al Daoud 2019). LightGBM also implements sparse optimization, multiple loss functions, regularization, bagging, early stopping, and efficient parallel training.

For comparison, we also developed prediction models based on other machine learning algorithms: RF (Svetnik *et al.* 2003), *k*NN (Song *et al.* 2017), ANN (Tadeusiewicz 2015), MLR (Vieira *et al.* 2016), AdaBoost (CAO *et al.* 2014), XGBoost (Ogunleye and Wang 2020), and SVM (Ben-Hur *et al.* 2008). RF is an ensemble learning method that combines multiple decision trees to improve model accuracy and generalization. It has been widely applied for classification as well as regression (Svetnik *et al.* 2003). *k*NN is another simple and efficient algorithm for both classification and regression tasks. *k*NN algorithm finds the *k*-nearest data points in a training dataset close to a given input data point, and then predict an output based on the majority vote or the average of the *k*-nearest neighbors (Song *et al.* 2017). ANN is an algorithm mimicking human brain learning and is composed of nodes and connections (Lancashire *et al.* 2009). The learning process of ANN is to find the best interconnections (weights) between nodes constituting the network topology. MLR is a statistical method used to model a linear relationship between a dependent variable and one or more independent variables. The objective of MLR is to find the best-fit line that represents the relationship between variables (Vieira *et al.* 2016). SVM is a supervised learning algorithm used for classification and regression analysis. Its fundamental concept is to identify a hyperplane that most effectively divides data points into distinct classes. The objective of the algorithm is to locate the decision boundary that optimizes the margin between the classes, which is defined as the gap between the hyperplane and the closest data points from each class (Ben-Hur *et al.* 2008). AdaBoost, known as Adaptive Boosting, is an ensemble method in machine learning. This algorithm initially assigns equal weights to all data points and constructs a model. It then increases the weights of misclassified points, emphasizing their importance in the subsequent model. This process continues until a lower error rate is achieved, leading to the training of multiple models (Cao *et al.* 2014). XGBoost (Extreme Gradient Boosting) uses gradient boosting, which adjusts the weights of misclassified data points to prioritize difficult-to-predict instances. This process leads to the creation of a strong ensemble model that provides accurate predictions for various tasks, such as classification and regression (Ogunleye and Wang 2020).

The 913 compounds were utilized as a training dataset. For evaluation, we conducted a 10-fold cross-validation with the training dataset. For model optimization, we performed parameter optimization since parameters often impact the accuracy of prediction models (Yang and Shami 2020). Parameters were optimized on the basis of their impact on model performance, GridSearchCV method was applied for parameters optimization and selected parameters and their range values investigated are listed in Supplementary Table S1 (Belete and Huchaiah 2022). An optimized model was evaluated on the independent dataset of 27 unique compounds.

### 2.3 Performance metrics

The quantitative model performance was measured based on two statistical criteria namely coefficient of determination (*R*^2^) and mean square error (MSE). They are defined as below:


(2) $$R^{2}=\;1-\;\frac{\sum_{i\;=\;1}^{n}{\left(y_{i}-{\hat{y}}_{i}\right)}^{2}}{\sum_{i\;=\;1}^{n}{\left(y_{i}-{\bar{y}}_{i}\right)}^{2}},$$

(3) $$\mathrm{MSE}=\;\frac{1}{n}\sum_{i\;=\;1}^{n}{\left(y_{i}-{\hat{y}}_{i}\right)}^{2}.$$

$y_{i}$ denotes a predicted logBB value, ${\hat{y}}_{i}$ denotes an actual logBB value, ${\bar{y}}_{i}$ is the mean of the actual values, and *n* is the amount of data. The *R*^2^ score close to +1 and MSE score close to 0 represent a higher accuracy and better performance. These metrics were used to evaluate our model and to compare the performances of publicly available models.

The qualitative model performance was calculated based on Matthew’s correlation coefficient (MCC), sensitivity, and specificity. They are defined as below:


(4) $$\mathrm{Sensitivity}=\;\frac{TP}{TP+FN},$$

(5) $$\mathrm{Specificity}=\;\frac{TN}{TN+FP},$$

(6) $$\mathrm{MCC}=\;\frac{TP\times TN-FP\times FN\;}{\sqrt{\left(TP+\;FP)(TP+\;FN)(TN+\;FP)(TN+\;FN\right)}},$$

where *TP* represents the number of true positives, *TN* represents the number of true negatives, *FP* represents the number of false positives, and *FN* denotes the number of false negatives.

## 3 Results and discussion

To advance the CNS drug discovery, it is essential to develop a cheap, fast, and accurate method to assess the BBB permeability of drug candidates. Computational prediction of BBB permeability would be an alternative method to the *in vitro* and *in vivo* methods. Though computational predictions are not perfect yet, they can reduce the number of drug candidates enough to afford experimental testing and allow high-throughput screening of a huge number of chemical compounds at a much faster speed. Thus, the challenge in CNS drug discovery is the development of an accurate BBB permeability prediction model.

In this study, we compiled the largest logBB dataset, to our knowledge, and used an efficient machine learning algorithm to build a more accurate quantitative model. Currently, most published BBB permeability prediction models are classification models that just determine whether a query compound is BBB-permeable or not (Castillo-Garit *et al.* 2017, Wang *et al.* 2018, Plisson and Piggott 2019, Singh *et al.* 2020). In CNS drug discovery, it is essential to predict the quantitative BBB permeability of chemical compounds, such as logBB since certain BBB-less-permeable drugs are still effective at low concentrations enough to be used as drug candidates. However, due to the limited data size of available logBB values, there are several quantitative BBB permeability prediction models (Sun 2004, Bayat *et al.* 2011, Wu *et al.* 2021) and only few of them are publicly available to access (Lee *et al.* 2004, Dyabina *et al.* 2016).

### 3.1 Overall flow of model construction

**Figure 1.**
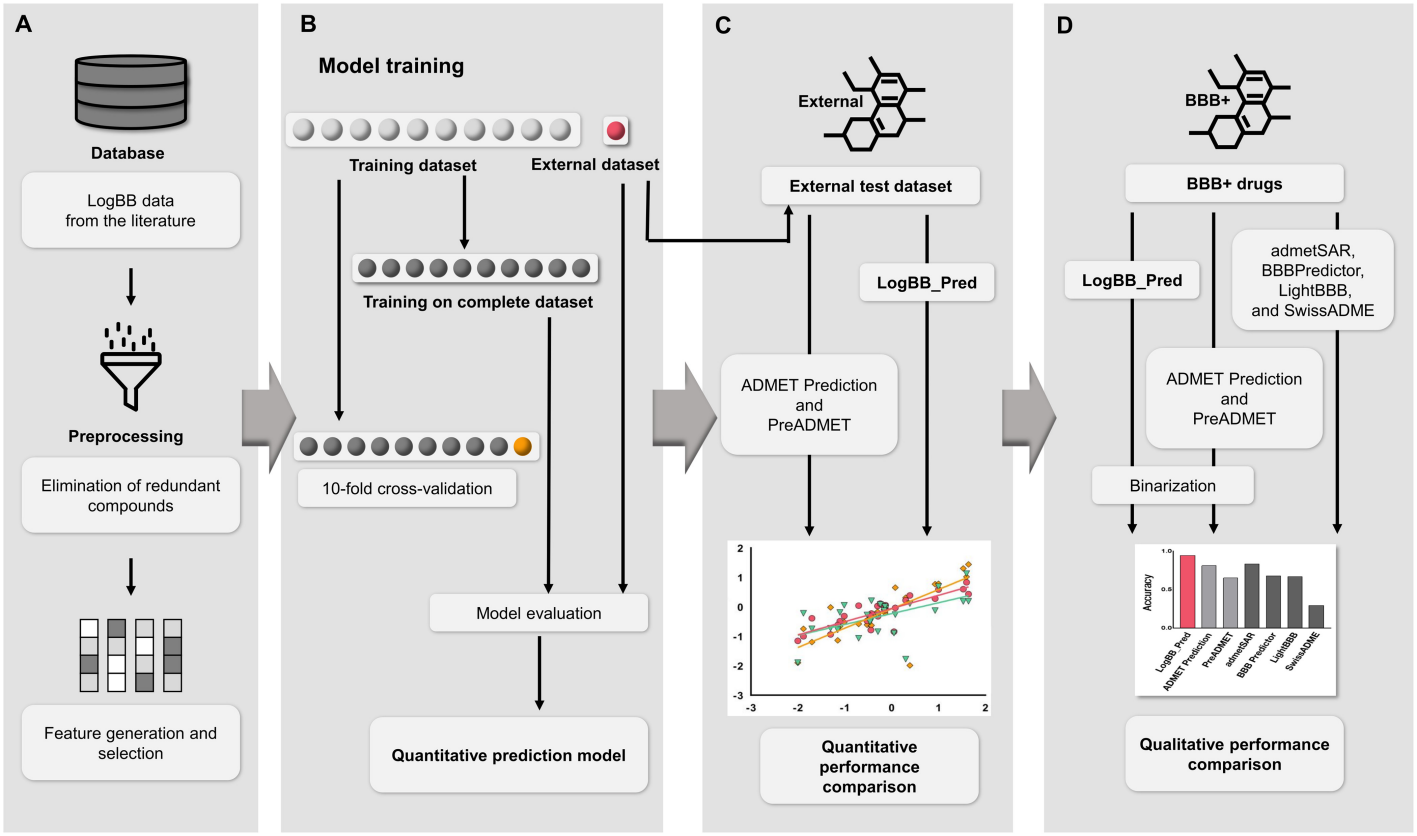
Schematic diagram of logBB prediction model construction. (A) LogBB dataset collection and features preprocessing. (B) Model was trained on a complete dataset and further evaluated on external dataset, and average model performance was also measured by applying 10-fold cross-validation. (C) Independent test dataset was used to compare the performance of our model with other publicly available quantitative models. (D) Binarized test dataset and additional BBB-permeable drug library were used to compare the performance of our model as a qualitative model with publicly available qualitative models.

The overall scheme to construct a quantitative logBB prediction model is illustrated in Fig. 1. Firstly, we collected the logBB values of chemical compounds from the various published literature and removed redundant chemicals to avoid biased or overfitted learning (Fig. 1A). To prepare features, we calculated the physical and chemical properties (descriptors) of the chemical compounds and discarded the descriptors with missing values. Redundant features were also removed based on PCC threshold. An optimal PCC threshold was determined by testing models built on various feature numbers, i.e. PCC threshold. Prediction models were developed based on various learning algorithms including LightGBM and trained models were cross validated (Fig. 1B). The final optimized model trained using LightGBM algorithm with different parameters were evaluated quantitatively on a test dataset and its performance was compared with other quantitative models (Fig. 1C). To investigate whether our model performs well as a classification model, the compounds in the test dataset were binarized into BBB-permeable and nonpermeable by a logBB threshold of −1 (Gao *et al.* 2017). The performance as a qualitative model was also compared with other qualitative models (Fig. 1D).

### 3.2 Data collection

In this study, as data size is one of the critical factors affecting the performance of machine learning models, we compiled 1276 logBB values of chemical compounds from the literature (Platts *et al.* 2001, Fu *et al.* 2005, Bayat *et al.* 2011, Muehlbacher *et al.* 2011, Carpenter *et al.* 2014, Shin *et al.* 2021, Tang *et al.* 2022), which is the largest logBB dataset, to our knowledge. Although we collected the largest dataset, it should be noted that logBB values were determined by different experimental methods or under different conditions, and, thus, the quality of the dataset can still be improved. However, this issue could be resolved only when a robust and high-throughput experimental method is used.

### 3.3 Data preprocessing

**Figure 2.**
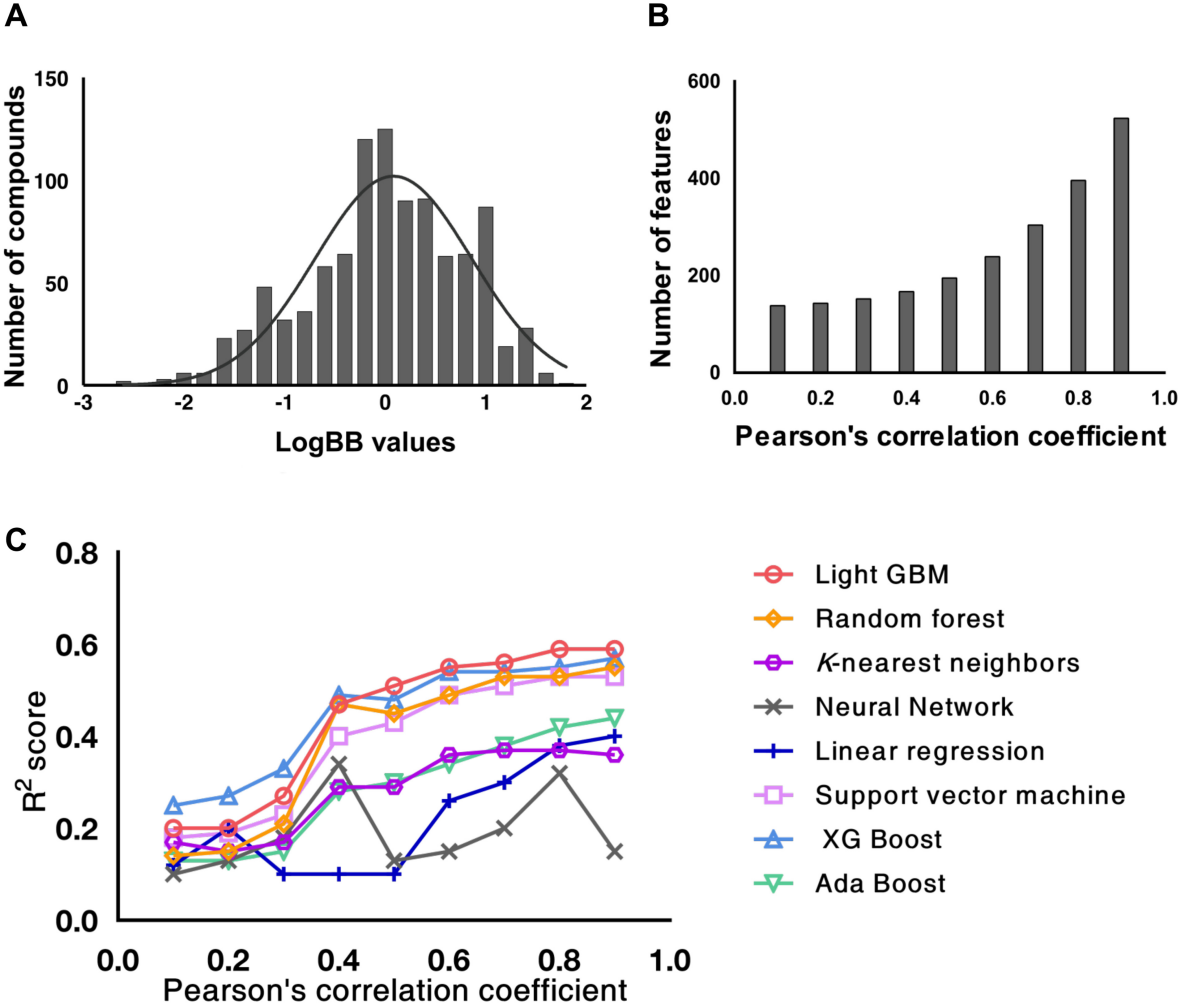
LogBB value distribution of collected data, and number of features, and performances with respect to the threshold of Pearson’s correlation coefficient. (A) Distribution of logBB values compiled in our dataset. (B) The number of selected features (*y* axis) when redundant features were removed based on a given threshold of Pearson’s correlation coefficient (*x* axis). (C) Ten-fold cross-validation results of the models trained using various learning algorithms with respect to various selected features based on Pearson’s correlation coefficient.

Since there might be similar chemical compounds in the collected logBB dataset, similar compounds were discarded based on Tanimoto similarity between chemicals to maintain the uniqueness of the compounds. Otherwise, the dataset may lead to a biased and overfitted model with abundant similar compounds. To calculate Tanimoto similarity, chemical compounds were firstly represented in SMILES format and then proceeded to Extended connectivity fingerprints (ECFPs) calculation using Dragon software (Mauri *et al.* 2006). The fingerprints, represented as 1024 bits of 0 or 1, were then used to calculate the similarity of two chemical compounds. The compounds with a similarity of over 0.85 were discarded from the dataset, which was a commonly accepted threshold to determine whether the two chemical compounds are similar or not (Macomber *et al.* 2015). Finally, 913 compounds were left in the logBB dataset and the distribution of these logBB values is shown in Fig. 2A.

In machine learning, numerical values (features) are required for training. Mordred was used to calculate the physical and chemical properties (descriptors), and those numerical values were used as features for machine learning. Mordred calculated 1650 2D and 3D molecular descriptors including molecular weight, lipophilicity (logP), number of rings, number of bonds, and number of atoms. Molecular descriptors are mathematical representation of molecular properties: 2D descriptors provide information regarding size, shape, and electronic distribution, and 3D descriptors describe the 3D conformation of a molecule, such as intramolecular bonding (Nettles *et al.* 2006). A total of 1650 descriptors were initially considered for the analysis. Those with missing values were removed from the dataset, leaving a subset of informative descriptors.

Like the similarity between chemicals, there may be similar features that have similar impact on model performance. We calculated pairwise PCC values between features, and between feature and logBB. If a pair of features has a greater correlation than a threshold, one with lower correlation with logBB was discarded. The optimal PCC threshold was 0.8 when we evaluated the effect of various PCC thresholds on performance.

### 3.4 Cross-validation with training dataset

We constructed models based on different learning algorithms (LightGBM, RF, *k*NN, MLR, SVM, AdaBoost, XGBoost, and ANN) and cross-validated them in 10-fold. Firstly, we set a PCC threshold and selected features. The number of features with respect to PCC threshold is shown in Fig. 2B. Once features were selected, six different models using different learning algorithms were constructed using 90% of the training dataset and then evaluated on the remaining 10% of the data. This model construction and evaluation were iterated 10 times and averaged performance values were obtained. The cross-validation results with respect to various feature numbers, i.e. PCC threshold, are shown in Fig. 2C. LightGBM outperformed other algorithms in terms of *R*^2^ score when trained with the features extracted using a PCC threshold of 0.8 (Fig. 2C**)**.

LightGBM has many advantages such as faster training speed, higher efficiency, and better accuracy, and, thus, it outperforms existing boosting frameworks in terms of accuracy (Al Daoud 2019). Another advantage is the employment of Gradient-Based One-Side Sampling and Exclusive Feature Bundling techniques, which allows handling a large number of data instances and data features, respectively, and therefore avoiding overfitting problems (Zhang *et al.* 2019). In addition, the algorithm supports an exclusive feature bundling to reduce the dimensionality of a dataset, and thereby making it faster and more efficient (Al Daoud 2019).

The evaluation revealed that the model trained using LightGBM algorithm with 396 informative features selected with a PCC threshold of 0.8 demonstrated better prediction performance in terms of *R*^2^ score compared with other algorithms and other numbers of features. The resulting average MSE of LightGBM model was 0.22 and its *R*^2^ score was 0.59 (Table 1). During the cross-validation, parameters of the algorithms were investigated to optimize the models since parameter optimization can improve model accuracy (Huang 2020). The investigated parameters of LightGBM are listed in Supplementary Table S1 along with their searched value ranges and selected optimal parameter values.

**Table 1.** Performance comparison of our model with publicly available quantitative models.

	Model	*R* ^2^	MSE	Reference
Cross-validation	Our model (LogBB_Pred)	0.59	0.22	This study
Independent evaluation	Our model (LogBB_Pred)	0.61	0.36	This study
	ADMET prediction service	0.56	0.41	Dyabina *et al.* (2016)
	PreADMET	0.3	0.66	Lee *et al.* (2004)

### 3.5 Performance comparison with other quantitative models

**Figure 3.**
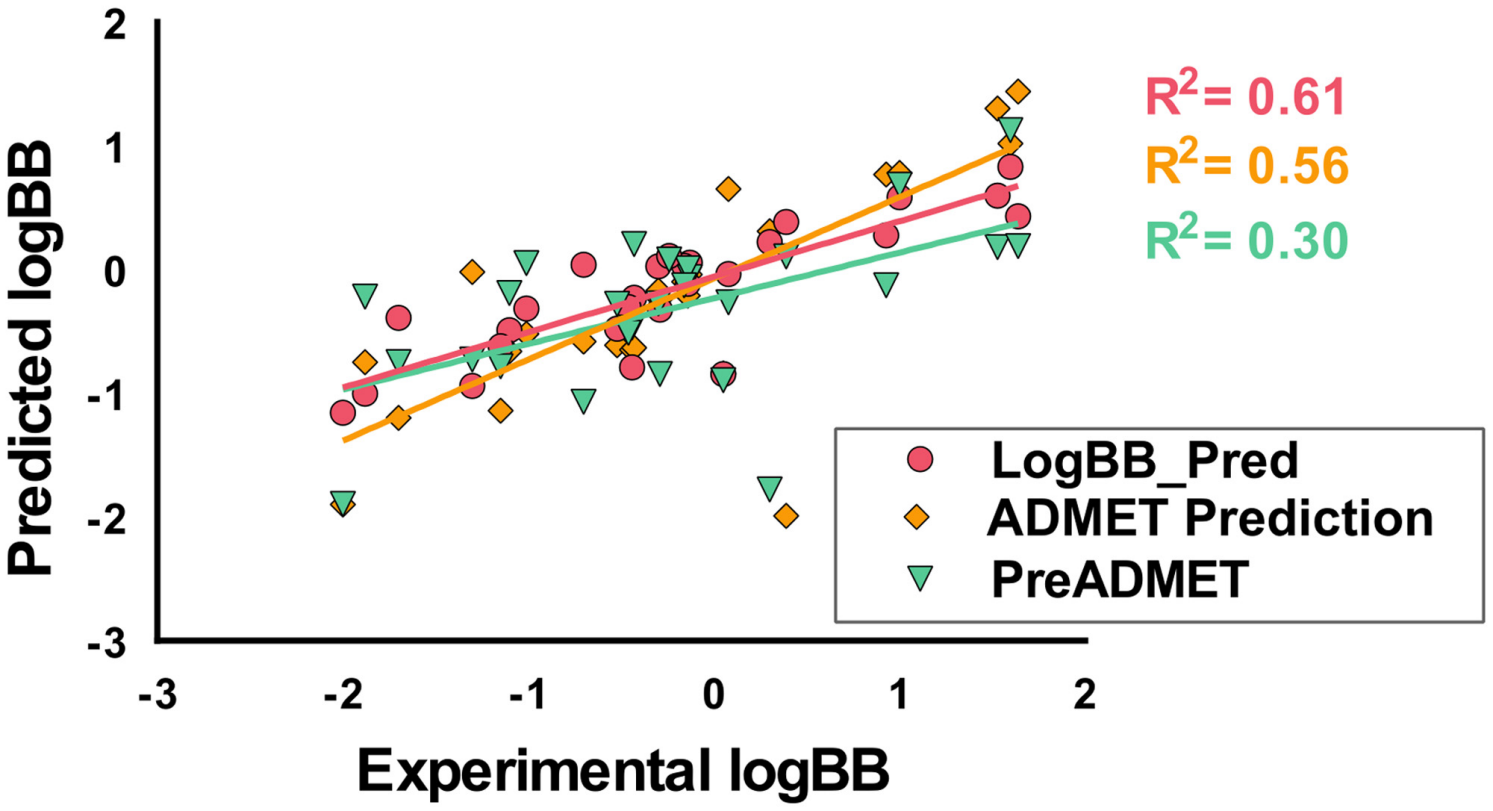
Performance comparison with other quantitative models. The predicted logBB values on an independent test dataset by our model and by other publicly available quantitative models. The predicted logBB values by our model are marked in red circles, those predicted by ADMET Prediction Service are marked in orange squares, and those predicted by PreADMET are marked in green triangles.

The LightGBM model was further evaluated using an independent dataset collected separately for an unbiased model validation. The model achieved an *R*^2^ score was 0.61 and MSE of 0.36 (Table 1 and Fig. 3), indicating that our model can be used as a highly accurate tool for predicting the potential blood–brain barrier permeability of query compounds. Therefore, it can also be used for screening large chemical compounds for CNS drug candidates.

The performance of our model was compared with those of publicly available quantitative BBB prediction models: ADMET Prediction Service (Dyabina *et al.* 2016) and PreADMET (Lee *et al.* 2004). As publicly available quantitative models are few, we could compare ours with only those two models. The compounds included in the test dataset were queried to the public models to predict their logBB values, and the predicted values are shown in Fig. 3 and their performances are shown in Table 1. It should be noted that the compounds used to train the model served at ADMET Prediction Service and PreADMET were not known, the compounds included in the test dataset might be used for the training of the models. Nonetheless, our model had a higher *R*^2^ score and smaller MSE than other models. The *R*^2^ scores of ADMET Prediction Service and PreADMET were 0.56 and 0.30, respectively. The MSE scores of the two models were 0.41 and 0.66, respectively. Consequently, our model can predict logBB values of query compounds more accurately and reliably.

### 3.6 Performance comparison with other qualitative models

To date, many BBB qualitative (classification) models, that predict whether a query molecule is BBB-permeable or not, have been published and some of them are publicly available to access. Thus, we compared the performance of our model with those of the available BBB qualitative models to investigate whether our quantitative model can also operate as a qualitative model and outperform conventional qualitative models.

The test dataset used to compare the performance of quantitative models was also used to compare the performances of qualitative models. To make our quantitative model operate as a qualitative model, compounds with a predicted logBB over the cutoff of −1.0 was categorized as BBB-permeable while those below the cutoff were classified as BBB-nonpermeable (Kunwittaya *et al.* 2013, Dyabina *et al.* 2016).

Our model achieved an accuracy of 85%, MCC of 0.60, and a positive predictive value (PPV) of 1.0, when it was used as a qualitative model on the independent test dataset (Table 2). The high MCC value represents that our model can accurately classify both BBB-permeable compounds and BBB-nonpermeable compounds. In addition, the high PPV value represents that our model can accurately identify compounds capable of crossing through BBB. The performance of our model was comparable with the best qualitative model (admetSAR) investigated in this study (Table 2), even though our model was developed as a quantitative model. In addition, the PPV of admetSAR was only 0.66, which means that only 66% of the admetSAR-suggested compounds are BBB permeable, while our model was 100%. This is important in drug discovery to find potential drug candidates and to avoid unnecessary experiments. Other qualitative models did not show better performance results than ours. Consequently, our model can be used not only to predict absolute logBB values but also to efficiently classify drug compounds into BBB-permeable or BBB-nonpermeable based on conventional logBB cutoff.

**Table 2.** Performance comparison of our model with publicly available qualitative models.

Dataset	Independent test dataset^a^				
Model	Our model (LogBB_Pred)	admetSAR	LightBBB	SwissADME	BBB predictor
Accuracy (%)	0.85	0.85	0.70	0.70	0.70
MCC	0.6	0.65	0.42	0.29	0.15
Sensitivity	0.42	0.85	0.70	0.57	0.28
Specificity	0.99	0.66	0.74	0.75	0.85
NPV^b^	0.83	0.94	0.45	0.44	0.80
PPV^c^	1	0.66	0.89	0.22	0.77
URL	http://ssbio.cau.ac.kr/software/logbb_pred	http://lmmd.ecust.edu.cn/admetsar2	http://ssbio.cau.ac.kr/software/BBB	http://www.swissadme.ch/	https://www.cbligand.org/BBB/index.php
Reference	This study	Yang *et al.* (2019)	Shaker *et al.* (2021)	Daina *et al.* (2017)	Liu *et al.* (2014)

a The independent test dataset used for the comparison of quantitative models was also used for qualitative model evaluation.

b Negative predictive value: (number of true negatives)/(number of true negatives + number of false negatives).

c Positive predictive value: (number of true positives)/(number of true positives + number of false positives).

**Figure 4.**
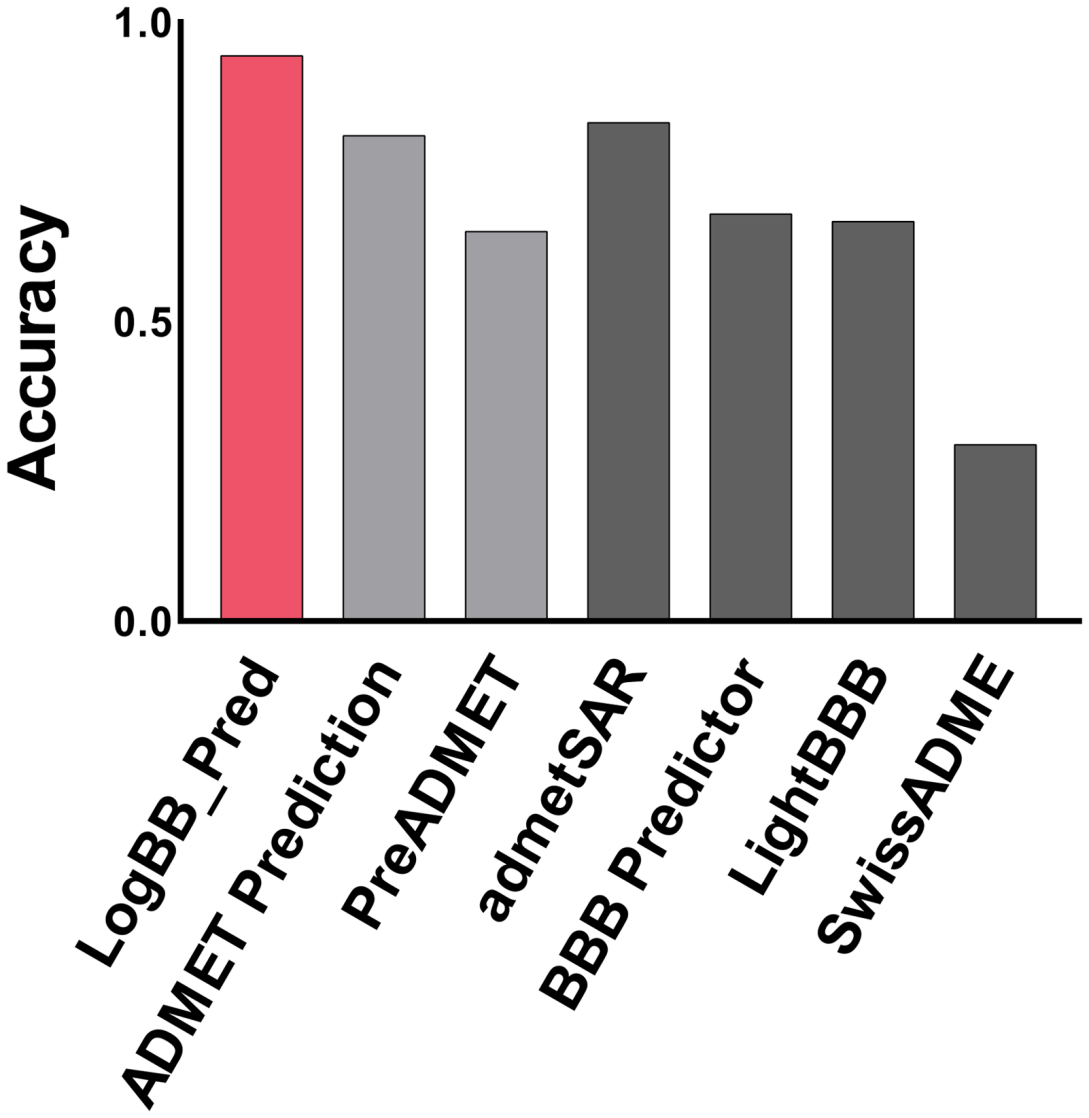
Performance comparison of qualitative models with a real-world CNS drug screening library. Three hundred and ninety-six compounds available from MCE company were used for model performance comparison. It should be noted that all the compounds in the library were BBB-permeable. The prediction accuracies of the quantitative models are shown in light gray color, and those of qualitative models are shown in dark gray color.

For further comparison, we also evaluated the qualitative models on the CNS drug screening library containing only BBB-permeable chemical compounds, obtained from MedChemExpress (https://www.medchemexpress.com/). The compounds included in our dataset or those similar to the compounds included in our dataset in terms of Tanimoto similarity were discarded. As a result, we obtained 396 BBB-permeable compounds. Our model outperformed the other qualitative models (Fig. 4). Our model achieved an accuracy of 97% while admetSAR achieved 83%, LightBBB achieved 67%, BBB Predictor achieved 67%, and SwissADME achieved 29%. We also evaluated the quantitative models (ADMET Prediction Server and PreADMET) on the CNS drug screening library with the same binarization of predicted logBB values as ours. They achieved accuracies of 81% and 65%, respectively. Comparing a quantitative model with a qualitative model poses inherent challenges. Notably, to our knowledge, we complied the largest logBB dataset and which was used to develop our quantitative model (LogBB_Pred). The larger dataset could be one of the factors for the improved performance of our model. These results indicate that our model is able to accurately predict BBB-permeable compounds even in a real-world drug screening library and would be practically used for CNS drug screening.

### 3.7 Web server construction

**Figure 5.**
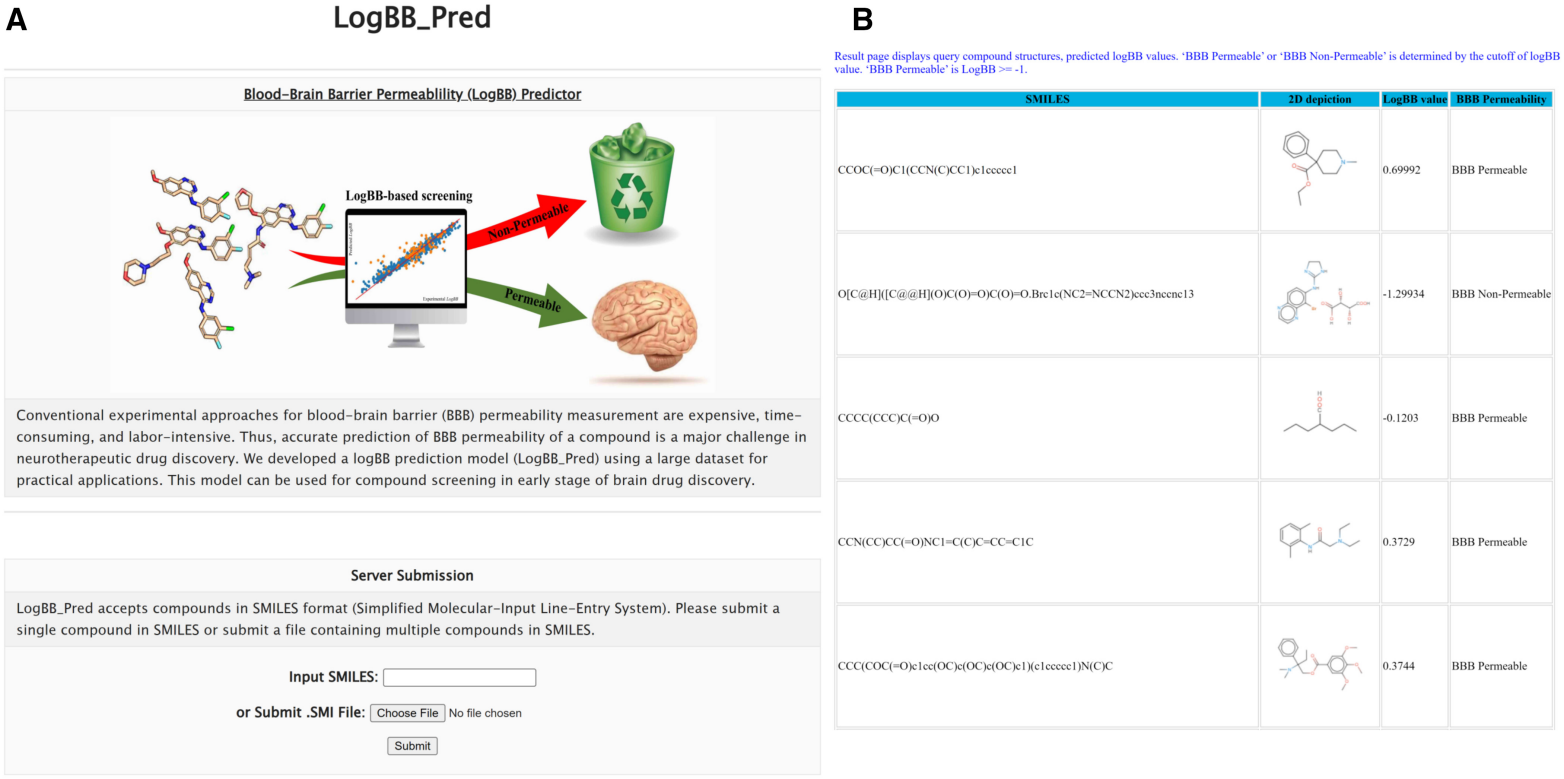
User interface of our LogBB_Pred web server. (A) Input interface where a user can submit a query compound in SMILES format or upload a file containing multiple compounds in the format of SMILES. (B) Prediction result page. The structure and predicted logBB value are displayed. “BBB Permeable” means its predicted logBB ≥ −1.

Developed prediction models should be freely accessible to drug developers, medicinal chemists, and other researchers to advance CNS drug discovery. To share our model, we constructed a web server that accepts a compound, or a list of compounds represented in a SMILES format and returns predicted logBB values (Fig. 5). The server is accessible via http://ssbio.cau.ac.kr/software/logbb_pred/.

## 4 Conclusion

Experimental methods to measure logBB values are costly and low throughput, thus making BBB permeability assessment a bottleneck in CNS drug discovery. In this study, we developed a quantitative model (LogBB_Pred) to predict an absolute logBB value of a query molecule, which showed superior performance over conventional prediction models. Our model can accurately identify which molecules are potentially BBB-permeable, and accurately predict what their logBB values are. Therefore, our model can be used for practical virtual screening of a large number of chemical compounds to find CNS drug candidates as an alternative to experimental methods and consequently facilitate the advance of CNS drug discovery.

## Supplementary Material

btad577_Supplementary_Data
