# Supplementary material for: A machine learning-based quantitative model (LogBB_Pred) to predict the blood–brain barrier permeability (logBB value) of drug compounds
Source: Bioinformatics. 2023 Sep 15;39(10):btad577. doi: 10.1093/bioinformatics/btad577 (PMC10560102; doi:10.1093/bioinformatics/btad577)
Supplement: btad577_Supplementary_Data [file btad577_supplementary_data.docx]

**Supplementary Information**

**Supplementary Table S1.** Parameters investigated and selected optimal parameter values.

| **Tuned parameters** | **Parameter search space** | **Optimal parameter value** |
| --- | --- | --- |
| n_estimators | 50 – 700 | 100 |
| num_leaves | 5 – 30 | 15 |
| max_depth | 5 – 25 | 10 |
| min_data_in_leaf | 10 – 15 | 15 |
| bagging_fraction | 0.2 – 0.8 | 0.6 |
